# Distribution and correlates of plantar hyperkeratotic lesions in older people

**DOI:** 10.1186/1757-1146-2-8

**Published:** 2009-03-30

**Authors:** Martin J Spink, Hylton B Menz, Stephen R Lord

**Affiliations:** 1 Musculoskeletal Research Centre, Faculty of Health Sciences, La Trobe University, Bundoora, Victoria 3086, Australia; 2 Prince of Wales Medical Research Institute, Randwick, New South Wales 2031, Australia

## Abstract

**Background:**

Plantar hyperkeratotic lesions are common in older people and are associated with pain, mobility impairment and functional limitations. However, little has been documented in relation to the frequency or distribution of these lesions. The aim of this study was to document the occurrence of plantar hyperkeratotic lesions and the patterns in which they occur in a random sample of older people.

**Methods:**

A medical history questionnaire was administered to a random sample of 301 people living independently in the community (117 men, 184 women) aged between 70 and 95 years (mean 77.2, SD 4.9), who also underwent a clinical assessment of foot problems, including the documentation of plantar lesion locations, toe deformities and the presence and severity of hallux valgus.

**Results:**

Of the 301 participants, 180 (60%) had at least one plantar hyperkeratotic lesion. Those with plantar lesions were more likely to be female (χ^2 ^= 18.75, *p *< 0.01; OR = 2.86), have moderate to severe hallux valgus (χ^2 ^= 6.15, *p *< 0.02; OR = 2.95), a larger dorsiflexion range of motion at the ankle (39.4 ± 9.3 *vs *36.3 ± 8.4°; *t *= 2.68, *df *= 286, *p *< 0.01), and spent more time on their feet at home (5.1 ± 1.0 *vs *4.8 ± 1.3 hours, *t *= -2.46, *df *= 299, *p *= 0.01). No associations were found between the presence of plantar lesions and body mass index, obesity, foot posture, dominant foot or forefoot pain. A total of 53 different lesions patterns were observed, with the most common lesion pattern being "roll-off" hyperkeratosis on the medial aspect of the 1^st ^metatarsophalangeal joint (MPJ), accounting for 12% of all lesion patterns. "Roll-off" lesions under the 1^st ^MPJ and interphalangeal joint were significantly associated with moderate to severe hallux valgus (*p *< 0.05), whereas lesions under the central MPJs were significantly associated with deformity of the corresponding lesser toe (*p *< 0.05). Factor analysis indicated that 62% of lesion patterns could be grouped under three broad categories, relating to medial, central and lateral locations.

**Conclusion:**

Plantar hyperkeratotic lesions affect 60% of older people and are associated with female gender, hallux valgus, toe deformity, increased ankle flexibility and time spent on feet, but are not associated with obesity, limb dominance, forefoot pain or foot posture. Although there are a wide range of lesion distribution patterns, most can be classified into medial, central or lateral groups. Further research is required to determine whether these patterns are related to the dynamic function of the foot or other factors such as foot pathology or morphology.

## Background

Hyperkeratotic lesions (calluses and corns) are highly prevalent in community dwelling older people, affecting 33 to 68% of those aged over 65 years [1-4]. Plantar lesions are frequently painful and are associated with reduced walking speed, impaired balance and difficulty in ascending and descending stairs [5], resulting in disability and reduced independence in older people [6]. An indication of the prevalence and impact of hyperkeratotic lesions in the community on the podiatric workforce is that lesion debridement accounts for up to 75% of podiatrist's workload [7] and that 84% of people seeking treatment for hyperkeratotic lesions will visit a podiatrist [8].

Hyperkeratosis is the result of abnormal mechanical stresses on the skin which stimulate overactivity of the keratinisation process. This causes accelerated proliferation of epidermal cells and a decreased rate of desquamation, resulting in hypertrophy of the stratum corneum [9]. The increased thickness results in a greater volume of skin through which mechanical stresses can be distributed. This natural process of symptom-free hyperkeratosis (*physiological hyperkeratosis*) helps to protect the skin and soft tissue layers from mechanical injury. Hyperkeratosis, however, becomes pathological when the keratinised material builds up sufficiently to cause tissue damage and pain, possibly through the release of inflammatory mediators [10] or due to the pressure of the central keratin plug on underlying nerves [11].

Plantar hyperkeratotic lesions are most commonly found under the metatarsophalangeal joints (MPJs) [11], and it has been identified in a number of studies that they develop in areas of elevated plantar pressure [7,12-14]. The largest study conducted on older people involved 292 participants and reported significant increases in plantar pressure under the callused regions of the plantar forefoot, with the exception of the 1^st ^MPJ [14]. The proposed association between elevated pressures and plantar hyperkeratotic lesions has led to some authors suggesting that there are characteristic patterns of lesion formation related to different foot types [15]. However, such associations have not been confirmed with objective data, and it is likely that lesion distribution patterns are also influenced by other variables, such as bodyweight [16], footwear [17], dominant foot [18] and toe deformities [19].

There have been four studies reporting on prevalence and distribution of plantar hyperkeratotic lesions [14,18,20,21]. The only study focused on older people (292 participants, mean age 77.6 years) reported the most common site to be the 1^st ^MPJ, followed by the 2^nd ^MPJ and then the hallux [14]. A study of 319 podiatry patients aged 20 to 99 years (mean age not reported) identified the 2^nd ^MPJ (36%) as the most common pattern for hyperkeratotic lesions, followed by the 1^st ^MPJ (27%) and the 5^th ^MPJ (13%) [21]. A study on 115 male runners (mean age 29.8 years) reported similar findings, with the 2^nd ^MPJ (32%) being the most common location, followed by the 1^st ^MPJ (23%) and the 5^th ^MPJ (13%) [18]. Finally, a study of 243 podiatry patients (mean age not reported) found hyperkeratotic lesions under the 2^nd^, 3^rd ^and 4^th ^MPJs to be the most common location (14%), followed by the 2^nd ^MPJ alone (10%) and both the 1^st ^and 5^th ^MPJs (8%) [20].

Although these studies have provided useful insights into the most common locations of plantar lesions, the underlying reasons for these patterns were not explored in detail. Therefore, the aims of this study were to evaluate the distribution of plantar hyperkeratotic lesions in a large sample of older people and to explore associations between the presence of lesions and physical characteristics (gender, obesity and dominant foot) and foot characteristics (foot posture, hallux valgus, lesser toe deformity and ankle flexibility). These variables were chosen as they could all be simply and non-invasively measured and are thought to be associated with callus growth through their influence on plantar pressure. We also investigated the relationship between the presence of forefoot callus and forefoot pain, as pain is the most common reason for people to seek medical care and has been associated with decreased ability to perform activities of daily living, problems with balance and gait and increased risk of falls [22]. We hypothesised that calluses would be more common in women, and would be significantly associated with obesity, foot pain, foot deformity (hallux valgus and lesser toe deformity) and reduced ankle joint range of motion.

## Methods

### Participants

The study population was derived from a larger study of risk factors for falls, and comprised men and women aged 70 years and over living in private households in the eastern suburbs metropolitan area of Sydney, New South Wales, Australia. These people were randomly drawn from the state electoral roll and initially contacted by letter and asked to participate in the study. Individuals were invited to the Prince of Wales Medical Research Institute for assessment. Potential participants were excluded from the study if they had minimal English language skills, were blind, or had a Mini Mental State Examination Score (MMSE) less than 24 [23]. Transport was provided for those who could not make their own way to the study site in order to maximise the participation rates of older people with mobility limitations. Of the 1,080 people initially contacted by letter and/or telephone, 329 (30.5%) agreed to participate and of these, 301 (117 men, 184 women) aged 70 to 95 years (mean = 77.2, SD = 4.9) met the inclusion criteria and attended an assessment appointment. The study was approved by the Human Ethics Committee, University of New South Wales and informed consent was obtained from all participants.

When compared with data from the national census and health survey for Australians aged over 70 living at home, the study group differed as follows: a higher proportion (61.1 *vs *56.5%) were female; a higher proportion (70.1 *v*s 46%) were aged 70–79 years, a higher proportion (74.8 *vs *66.7%) were Australian born and a higher proportion (41.9 *vs *32.7%) were living alone.

### Foot assessment

Age and medical history were determined by an interviewer-administered questionnaire including the amount of time spent on their feet at home performing housework, self-care or walking around the house and self reported dominant foot, identified by the response to the question ''Which foot would you use to kick a ball with?''. The presence and severity of hallux valgus was determined using the Manchester Scale [24]. This instrument consists of standardised photographs of feet with four degrees of hallux valgus – none (score = 0), mild (score = 1), moderate (score = 2) and severe (score = 3) which were matched to the participant's feet. The grading of hallux valgus using this tool is highly correlated with angular hallux valgus measurements obtained from foot radiographs [25]. Presence of calluses, corns and lesser digital deformity (hammertoes and claw toes) were documented on a foot map. Forefoot pain was also recorded as present or not using a foot map. Arch height was assessed by measuring the height of the navicular tuberosity in millimetres while the subject was fully weightbearing. This score was then corrected for differences in foot size by dividing it by the length of the foot [26]. Navicular height has previously been reported to have high intratester reliability and is closely correlated with navicular height determined from lateral radiographs [27]. Ankle flexibility was measured in degrees using a modified version of the weightbearing lunge test. The lateral malleolus and head of the fibula were first located and marked with an ink pen. Participants then stood with their right foot placed alongside a vertically-aligned clear acrylic plate inscribed with 2° protractor markings, and were instructed to take a comfortable step forward with the left leg. In this position, participants were instructed to bend their knees to squat down as low as possible, without lifting the right heel from the ground and while keeping the trunk upright. Participants leant on a bench placed alongside them at waist height to support their bodyweight. The position of the fibular head was marked on the clear acrylic plate, and the angle formed between the lateral malleolus and the fibular head was measured. The test was completed three times, and the average score documented as the test result [27]. High intra-observer reliability for both the navicular height (ICC = 0.64) and ankle flexibility (ICC = 0.87) when performed on older people has been established previously for the testers involved in this study [27].

### Statistical analysis

All statistical tests were conducted using SPSS Release 14 for Windows (SPSS Inc, Chicago, IL, USA). Associations and comparisons between participants with and without hyperkeratotic lesions were determined using the chi-square statistic and odds ratios (for dichotomous variables) and independent samples t-tests (for continuous variables), respectively. Factor analysis, a data reduction technique, is used in an exploratory fashion to reveal patterns among the inter-relationships of the items [28]. In this study it was used to determine whether the large number of lesion distribution combinations could be collapsed into smaller groups. In order to determine the suitability of the data for factor analysis, the Kaiser-Meyer-Olkin Measure of Sampling Adequacy (KMO) and Bartlett's Test of Sphericity were calculated. KMO indicates whether or not the variables are able to be grouped into a smaller set of underlying factors. Values for KMO range from 0 to 1, with values over 0.5 indicating an acceptable and increasing degree of common variance. Bartlett's test of sphericity is another measure of whether the variables in the population matrix are correlated. It is reported as a significance level with lower significance levels indicating a stronger correlation between the variables [29]. A principal component analysis was then performed. A three component solution was extracted using the Kaiser-Guttman rule (eigenvalues > 1.0), and varimax rotation was performed to minimize the complexity of loadings for each component.

## Results

### Characteristics of the study population

The characteristics of the study population are shown in Table 1. Comparisons between those with and without plantar lesions are shown in Table 2. Of the 301 participants, 181 (60%) had at least one plantar hyperkeratotic lesion. Those with plantar lesions were more likely to be female (χ^2 ^= 18.75, *p *< 0.01; odds ratio [OR] = 2.86), have moderate to severe hallux valgus (χ^2 ^= 6.15, *p *< 0.02; OR = 2.95) and have a larger dorisflexion range of motion at the ankle (39.4 ± 9.3 *vs *36.3 ± 8.4°, *t *= 2.68, *df *= 286, p < 0.01). However, there were no differences (*p *> 0.05) between the groups in relation to BMI, obesity, foot posture or dominant foot.

**Table 1:** Sample characteristics, including prevalence of major medical conditions. Numbers are n (%) unless otherwise stated.

Condition	
Age – years (SD)	77.2 (4.9)
Women	184 (61)
Body mass index – mean kg/m^2 ^(SD)	25.9 (4.1)
Obese (BMI > 30)	49 (16)
Diabetes	16 (5.3)
Parkinson's disease	5 (1.7)
Hearing problem	108 (35.9)
Peripheral vascular disease	45 (15)
Stroke	9 (3)
Transient ischaemic attack	12 (4)
Heart disease	71 (32.6)
Hypertension	134 (44.5)
Incontinence	63 (20.9)
Osteoarthritis	174 (57.8)
Hip fracture	8 (2.7)

**Table 2:** Characteristics of participants with and without hyperkeratotic lesions.

Condition	No hyperkeratotic lesions (n = 121)	Hyperkeratotic lesions (n = 180)
Age – years (SD)*	78.1 (5.0)	76.6 (4.7)
Women – n (%)**	56 (46)	128 (71)
Body mass index – kg/m^2 ^(SD)	25.6 (3.9)	26.2 (4.2)
Obese – BMI > 30 n (%)	15 (12)	34 (19)
Navicular height – mm (SD)†	18.0 (5.9)	18.0 (5.9)
Moderate to severe hallux valgus (right foot) – n (%)**	11 (8)	39 (24)
Moderate to severe hallux valgus (left foot) – n (%)*	17 (11)	35 (24)
Ankle range of motion (sagittal plane) - degrees (SD)**	36.3 (8.4)	39.4 (9.3)
Time spent on feet during the day – hours (SD)*	4.8 (1.3)	5.1 (1.0)

*p < 0.05, **p < 0.01

† normalized for foot length

### Patterns of plantar lesions

**Figure 1:**
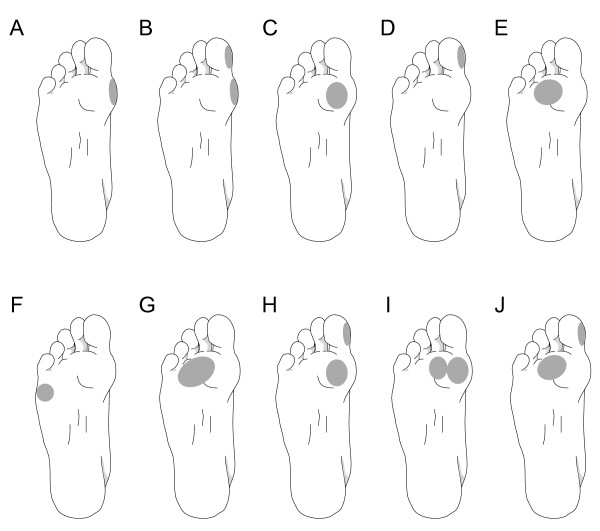
**Most common plantar hyperkeratotic lesions patterns-n (%)**. A: 40 (12%), B: 40 (12%), C: 38 (12%), D: 27 (9%), E: 24 (8%), F: 14 (5%), G: 9 (3%) H: 7 (2%), I: 7 (2%), J: 6 (2%).

Of the 604 feet, 308 (51%) had plantar hyperkeratotic lesions over at least one of the MPJs, or "roll-off" callus on the medial aspect of the 1^st ^MPJ or 1^st ^interphalangeal joint (IPJ). A total of 53 different lesions patterns were recorded. The ten most common patterns, which accounted for 62% of all lesion patterns, are shown in Figure 1.

### Correlates of plantar lesions

There were no gender differences in lesion patterns, with the exception of a higher prevalence in women for lesions over the medial aspect of the 1^st ^MPJ on both feet (χ^2 ^= 9.24, *p *< 0.01; OR = 2.32), the right 2^nd ^MPJ (χ^2 ^= 5.90, *p *= 0.02; OR = 2.28) and the medial aspect of the 1^st ^IPJ for the left foot (χ^2 ^= 19.15, *p *< 0.01; OR = 4.48). Moderate to severe hallux valgus was associated with "roll-off" lesions over the medial aspect of the 1^st ^MPJ joint for both right (χ^2 ^= 25.69, *p *< 0.01; OR = 5.39) and left foot (χ^2 ^= 28.31, *p *< 0.01; OR = 5.92) and the 1^st ^IPJ for the left foot (χ^2 ^= 4.55, *p *= 0.03; OR = 2.13). There was also a significant association between lesions under the MPJs and deformity of the corresponding toe for the 2^nd ^(χ^2 ^= 4.88, *p *= 0.03; OR = 2.01) and 4^th ^(χ^2 ^= 4.48, *p *< 0.03; OR = 3.07) MPJs on the right foot, and the 2^nd ^(χ^2 ^= 4.72, *p *= 0.03; OR = 2.15) and 3^rd ^(χ^2 ^= 7.34, *p *< 0.01; OR = 2.79) MPJs on the left foot. Those with plantar calluses also spent more time on their feet at home (5.1 ± 1.0 *vs *4.8 ± 1.3 hours, *t *= -2.46, *df *= 299, *p *= 0.01).

There were no associations between forefoot pain and presence of plantar lesions (either globally [*p *= 0.64] or specific to individual locations). Similarly, the total number of lesions did not differ between those who did and did not report forefoot pain (1.83 ± 2.3 *vs *2.0 ± 2.3; *p *= 0.55).

### Factor analysis of lesion patterns

The Kaiser-Meyer-Olkin Measure of Sampling Adequacy was found to be 0.6, which meets the recommended minimum value [28].

The Bartlett's Test of Sphericity was highly significant (χ^2 ^= 167.5, *p *< 0.001), supporting the suitability of the data for factor analysis [29]. Results of the factor analysis are shown in Table 3. A three-factor model was extracted which accounted for 62% of the total variance. Component 1 incorporated three lesion sites under the central forefoot (2nd, 3rd and 4th MPJs), component 2 incorporated three lesion sites under the medial forefoot (medial 1st IPJ, medial 1st MPJ and 1st MPJ), and component 3 incorporated two lesion sites under the central-lateral forefoot (4th and 5th MPJs).

**Table 3:** Component coefficients derived from the factor analysis.

	Component		
Lesion site	1	2	3

Medial 1^st ^IPJ	-	0.583	-
Medial 1^st ^MPJ	-	0.732	-
1^st ^MPJ	-	0.840	-
2^nd ^MPJ	0.840	-	-
3^rd ^MPJ	0.891	-	-
4^th ^MPJ	0.547	-	0.533
5^th ^MPJ	-	-	0.858

Factor loadings less than 0.5 have been omitted in order to improve clarity.

## Discussion

The purpose of this study was to evaluate the distribution of plantar hyperkeratotic lesions in a large sample of older people. Before discussing these findings, however, it needs to be acknowledged that the response rate of the study population was relatively low (30%). This is comparable to one of the previous studies on callus distributions, where the response rate was 29% [21]. Response rates for other callus distribution studies were either not stated [14] or the participants were obtained using convenience sampling [18,20]. Due to the study exclusion criteria, it is acknowledged that the majority of the participants were independent community-dwelling people and the findings may not be generalised beyond this group. Furthermore, it should be noted that the variations between the study sample and the national census data indicate this sample was biased towards Australian-born women under 80 years old living alone.

Sixty percent of the sample had at least one plantar hyperkeratotic lesion. This figure concurs with a number of other community-based studies of older people, which have reported prevalence rates ranging between 26 and 68% [1-4]. The higher prevalence observed in women is also consistent with previous studies [2,3] and is likely to be related to the wearing of shoes with an elevated heel and narrow toe box [17], although other factors such as the higher prevalence of hallux valgus in females [30] may also be responsible. Heel elevation increases the pressure borne by the metatarsal heads [31,32] and it has previously been demonstrated that older people who wear shoes that are too narrow or too short are more likely to have corns, lesser toe deformities and hallux valgus [17].

This is the first study to make a distinction between centrally located callus and callus at the plantar-medial aspect of the 1^st ^MPJ and IPJ, often referred to as "roll-off" callus. Interestingly, the most common lesion pattern found in this study was medial roll-off callus at the 1^st ^MPJ (13%), followed by medial roll-off callus at both the 1^st ^MPJ and 1^st ^IPJ (13%), then over the 1^st ^MPJ (12%). If roll-off callus is excluded, lesions under the 1st MPJ (28%) was the most common pattern, followed by under both the 2^nd ^and 3^rd ^MPJs (16%) then the 5^th ^MPJ (11%), which is similar to Menz et al [14], who found the most common site to be the 1^st ^MPJ, followed by the 2^nd ^MPJ and then the hallux in a sample of 292 older people.

The findings of this study differ to the three other studies on the distribution of plantar hyperkeratotic lesions [18,20,21]. Springett et al [21] and Grouios [18] found the 2^nd ^MPJ to be the most common location followed by the 1^st ^MPJ then the 5^th ^MPJ. In contrast, Merriman [20] found hyperkeratotic lesions under the 2^nd^, 3^rd^, and 4^th ^MPJs to be the most common, followed by the 2^nd ^MPJ alone and both the 1^st ^and 5^th ^MPJs. This study also included callus under the 1^st ^IPJ but did not report if it was roll-off callus or centrally located, and the prevalence was considerably lower compared to the current study. The differences between these results and those of the current study may be due to differences in participant characteristics, as the aforementioned studies generally involved younger people, or specific populations such as male runners [18] or people presenting for podiatric treatment [18,20]. Furthermore, it is unclear whether previous studies have excluded roll-off callus or included these lesions as either 1^st ^MPJ or 1^st ^IPJ callus. Even if roll-off callus is excluded, however, this study still had a much higher prevalence of lesions under the 1^st ^MPJ.

This may be due to the higher prevalence of hallux valgus in older people [33]. Furthermore, it has been reported that peak pressure in the older foot is higher under the medial forefoot area [34]. The predisposition to medially-located lesions is reflected in the results of the factor analysis (Table 3) which identified that the hyperkeratotic lesion distribution could be collapsed into three groups, essentially a medial, central and lateral grouping, with the medial group consisting of medial 1^st ^IPJ, medial 1^st ^MPJ and 1^st ^MPJ.

Although there is no previous evidence of callus being linked to range of motion in foot joints, our finding of a slightly *larger *range of ankle dorsiflexion in those with forefoot calluses is somewhat counter-intuitive, given that reduced ankle motion has been shown to increase forefoot loading in people with diabetes [35]. However, ankle flexibility is positively correlated with walking speed [36], and walking speed is in turn associated with higher forefoot pressures [37]. Therefore, it is possible that increased ankle flexibility in those with calluses is a marker of increased walking speed, which was not analysed in this study. Further research involving concurrent measurement of dynamic ankle motion and plantar pressures would help clarify this relationship.

We found no association between forefoot pain and the presence of plantar lesions. This observation is consistent with Garrow et al [38] and Menz and Morris [39], but contrasts to Benvenuti et al [6] and Menz et al [14], who found that older people with calluses were more likely to report foot pain. While this study did not record specific details on mobility such as the participants' physical activity levels or walking distances, they were asked to report the average time spent on their feet doing housework and self care around the home. Interestingly, the group with callus reported spending significantly more time on their feet. This could be interpreted as a potential *cause *of the plantar lesions (i.e. increased duration of weightbearing), or may simply indicate that the presence of non-painful callus does not interfere with activities of daily living.

We also found no association between plantar hyperkeratotic lesions and bodyweight, obesity, foot posture or dominant foot. Bodyweight has been shown to be a significant determinant of plantar pressure in older people [40] and increases in force and pressure under the foot when walking, particularly under the midfoot and metatarsal heads, have been observed in obese people [16,41]. The lack of an association between plantar lesions and bodyweight indicates that factors other than increased plantar pressure (such as soft tissue thickness, skin hydration and vascular status) may be responsible for the formation of hyperkeratotic lesions in older people. Similarly, it might be expected that foot posture, by altering plantar pressure distribution, would increase the likelihood of developing lesions under certain plantar regions. However, although flatter/more pronated feet and reduced range of motion of the ankle and 1^st ^MPJ have been demonstrated in older people [33], and higher plantar pressure have been shown in people with pes cavus [42], we found no significant association between foot posture and hyperkeratotic lesions. Although the inclusions of a broader array of foot posture measurements may have produced a different result, our findings suggest that obvious structural foot deformities such as hallux valgus and lesser toe deformities play a greater role in plantar lesion development in older people than foot posture.

Finally, it has been suggested that a greater mechanical demand is placed on a person's dominant side and may influence gait patterns, resulting in hyperkeratotic lesions [18,21]. While this has been shown to be case in one study on a younger, athletic sample [18], we found no association between dominant foot and callus formation, which concurs with the findings of Springett et al [21].

## Conclusion

Plantar hyperkeratotic lesions affect 60% of older people and are associated with female gender, hallux valgus, toe deformity, increased ankle flexibility and time spent on feet during the day, but are not associated with obesity, limb dominance, forefoot pain or foot posture. Although there are a wide range of lesion distribution patterns, most can be classified into medial, central or lateral groups. Further research is required to determine the most effective strategies for the prevention and treatment of these lesions.

## Competing interests

HBM is Editor-in-Chief of the *Journal of Foot and Ankle Research*. It is journal policy that editors are removed from the peer review and editorial decision making processes for papers they have co-authored.

## Authors' contributions

SRL and HBM conceived and designed the study. HBM conducted the statistical analysis. MJS compiled the data and drafted the manuscript and HBM contributed to the drafting of the manuscript. All authors read and approved the final manuscript.
